# Pulmonary Hypertension in Obese Mice Is Accompanied by a Reduction in PPAR-γ Expression in Pulmonary Artery

**DOI:** 10.3389/fendo.2021.701994

**Published:** 2021-09-06

**Authors:** Any Elisa de Souza Schmidt Gonçalves, Guilherme Zweig Rocha, Rodrigo Marin, Rafael Ludemann Camargo, Andrey dos Santos, Helison do Carmo, Dioze Guadagnini, Orlando Petrucci, Zenaide Providello Moysés, Vera Maria Cury Salemi, Alexandre Gabarra Oliveira, Mario José Abdalla Saad

**Affiliations:** ^1^Department of Internal Medicine, Faculty of Medicine, State University of Campinas, Campinas, Brazil; ^2^Department of Physical Education, São Paulo State University (UNESP), Rio Claro, Brazil; ^3^Heart Institute (InCor) do Hospital das Clínicas da Faculdade de Medicina da Universidade de São Paulo, São Paulo, Brazil

**Keywords:** obesity, insulin resistance, pioglitazone, PPAR-γ, high-fat (HF) diet

## Abstract

Obesity and insulin resistance (IR) are well-studied risk factors for systemic cardiovascular disease, but their impact on pulmonary hypertension (PH) is not well clarified. This study aims to investigate if diet-induced obesity induces PH and if peroxisome-proliferator-activated receptor (PPAR-γ) and/or endoplasmic reticulum (ER) stress are involved in this process. Mice were maintained on a high-fat diet (HFD) for 4 months, and IR and PH were confirmed. In a separate group, after 4 months of HFD, mice were treated with pioglitazone (PIO) or 4-phenylbutyric acid for the last month. The results demonstrated that HFD for at least 4 months is able to increase pulmonary artery pressure, which is maintained, and this animal model can be used to investigate the link between IR and PH, without changes in ER stress in the pulmonary artery. There was also a reduction in circulating adiponectin and in perivascular adiponectin expression in the pulmonary artery, associated with a reduction in PPAR-γ expression. Treatment with PIO improved IR and PH and reversed the lower expression of adiponectin and PPAR-γ in the pulmonary artery, highlighting this drug as potential benefit for this poorly recognized complication of obesity.

## Introduction

Pulmonary hypertension (PH) is a disease of the pulmonary circulation associated with an excessive proliferation of vascular cells that may induce alterations in the resistance of the pulmonary arteries (PAs), but systemic vessels are spared (1, 2). It may be idiopathic or familial, which are rare forms, but most often, PH is associated with more common diseases such as left heart failure, collagen vascular diseases, congenital heart disease, and AIDS, among others (1, 3). PH is defined by mean pulmonary artery pressure ≥25 mmHg at rest or 30 mmHg during exercise, measured invasively during right heart catheterization (4, 5), and presented a progressive increase in pulmonary vascular resistance, right heart failure, and death in most patients after 5 years since diagnosis (6, 7).

The molecular pathophysiology of PH has been studied in the past years and involves multiple molecular pathways and triggers including endoplasmic reticulum (ER) stress and signaling of Notch, mutations in the bone morpho-genetic protein receptor II (BMP-RII), hypoxia, viruses, tyrosine kinase receptors and signaling pathways, hypoxia-inducible factor-1a (HIF-1a), and peroxisome-proliferator-activated receptor (PPAR) (8–10). The lack of complete understanding of the mechanisms underlying PH limits the development of effective therapies.

Although obesity and insulin resistance (IR) are well-studied risk factors for systemic cardiovascular disease, their impact on PH is not well clarified. Zamanian and colleagues showed that patients with PH have widespread metabolic dysfunction, including IR, and also indicated that metabolic changes can occur due to inhibition of an essential transcription factor, PPAR-γ (11). Patients who develop PH have reduced expression of apoE and PPAR-γ in their lungs, and deficiency of both is directly linked to IR (12). On the other hand, Sutendra and collaborators considered the ER stress present in endothelial muscle cells of the pulmonary artery as a factor for the development of PH, placing ER stress as a common molecular hypotheses among IR and PH (13).

However, the role of obesity in inducing PH, and the molecular mechanism behind this process, is still scarce. In this regard, the aim of the present study was to investigate if diet-induced obesity is able to induce PH, and if so, whether PPAR-γ and/or ER stress are involved in this process.

## Methods

### Animals

Male C57BL6/J mice were provided by the State University of Campinas Central Breeding Center (Campinas, Brazil). Eight-week-old male C57BL6/J mice were maintained under specific pathogen-free conditions in a regimen of light/dark cycle of 12 h, and temperature set to 23°C ± 2°C. All experiments were conducted according to the “Guide for the Care and Use of Laboratory Animals of the Institute of Laboratory Animal Resources, US National Academy of Sciences” and were approved by the Ethics Committee (Comissão de Ética no Uso de Animais/Instituto de Biologia/Universidade Estadual de Campinas number 2837-1).

### Experimental Design

The animals were randomly divided into two groups with similar body weights, according to the diet that they were assigned to receive: control animals fed a standard rodent chow (8% fat, 26% protein, and 54% carbohydrate, as a percentage of total kcal) (CTL group) or a high-fat diet (55% of energy derived from fat, 29% from carbohydrates, and 16% from protein) (HFD group) (14) for 4 months. Food and water were available *ad libitum*. After 4 months of feeding, the animals underwent echocardiographic and hemodynamic measurements before sacrifice. After fasting, blood samples were collected and centrifuged at 3,000×*g* for 10 min at 4°C to obtain serum for the determination of basal insulin (Millipore, St. Charles, MO, USA) and adiponectin (Millipore, St. Charles, MO, USA) by ELISA. Glucose values were measured from the tail venous blood of all animals with a glucose monitor (Glucometer; Bayer, Tarrytown, NY, USA). HOMA-IR was calculated from the fasting concentrations of glucose (mg/dl) and insulin (mU/L) through the HOMA-IR index (HOMA-IR = fasting glucose × fasting insulin/405). The animals that developed PH (HFD) were treated for 30 days by oral gavage with PIO (20 mg/kg/day—an agonist of PPAR-γ) (15, 16) and 4-phenylbutyric acid (PBA) (500 mg/kg/day—an inhibitor of endoplasmic reticule stress) (17). CTL and HFD animals were treated with vehicle as an internal control (**Figure S1**).

### Intraperitoneal Glucose Tolerance Test

The mice fasted for 6 h before blood samples were collected. After the collection of a fasting sample (time 0), the mice received an injection of glucose (20% solution in saline) into the peritoneum. Blood glucose measurements were assessed through tail blood and repeated at 30, 60, 90, and 120 min after glucose challenge.

### Insulin Tolerance Test

The mice fasted for 6 h before blood samples were collected. After the collection of a fasting sample (time 0), the mice received one injection of insulin (1.5 IU/kg body weight) into the peritoneum. Blood glycemia was verified at 5, 10, 15, 20, 25, and 30 min. The constant rate for glucose disappearance (kITT) was calculated from the slope of the least square analysis of the blood glucose concentrations during the linear phase of decay.

### Tissue Extraction and Western Blotting

The mice fasted for 6 h before procedures. Briefly, the mice were anesthetized, and after assurance of loss of pedal and corneal reflexes, they were intraperitoneally injected with insulin (1 U/kg) or saline, and after 10 min, the pulmonary artery was extracted and homogenized in extraction buffer [10 mmol/L ethylenediaminetetraacetic acid (EDTA), 100 mmol/L Tris (pH 7.4), containing 100 mmol/L sodium pyrophosphate, 100 mmol/L sodium fluoride, 10 mmol/L sodium vanadate, 2 mmol/L phenylmethylsulfonyl fluoride (PMSF*)*, and 0.1 mg of aprotinin/ml, and 1% Triton-X 100]. The samples were centrifuged at 11,000 rpm and 4°C, and the supernatants were used.

### Western Blotting Analysis

The samples were treated with Laemmli sample buffer (100 mM dithiothreitol) and heated at 100°C for 5 min, after which they were subjected to sodium dodecyl sulfate–polyacrylamide gel electrophoresis (SDS-PAGE) in a Bio-Rad (Hercules, CA, USA) miniature slab gel apparatus (Mini-Protean). Protein transfer from the gel to nitrocellulose membranes was performed for 90 min at 120 V in a Bio-Rad Mini-Protean transfer apparatus (14). Nonspecific protein binding to the nitrocellulose was reduced by preincubating for 2 h in blocking buffer (5% non-fat dry milk, 10 mM Tris, 150 mM NaCl, and 0.02% Tween 20). The nitrocellulose blot was incubated overnight at 4°C with the following antibodies: anti-Akt (SC-81434 mouse monoclonal), anti-phospho-Akt (SC-7985 rabbit polyclonal), anti-phospho-JNK (SC-6254 mouse monoclonal), anti-PPAR-γ (SC-7273 mouse monoclonal), and anti-β−tubulin (SC-5274 mouse monoclonal). The secondary antibody linked to a peroxidase molecule reacted with the chemiluminescence solution (ClarityTM Western ECL substrate kit, BioRad^©^), and the membranes were developed in photodocumentation (Gel Doc™ XR, BioRad^©^), generating digital files. Later, the images were analyzed using the software ImageLab (v. 5.2.1 build 11, Bio-Rad ^©^ Laboratories). The Akt phosphorylation levels were normalized by total Akt levels.

### Hemodynamic Measurements

The mice were anesthetized with inhalation of isoflurane 2% (1 ml/ml), intubated, and mechanically ventilated (115 breaths per minute, FiO_2_ = 1). A micromanometer pressure catheter (SPR 671, 1,4F 56 cm, Mikro-Tip^®^, Millar) was placed into the right ventricle for intraventricular arterial pressure monitoring (18). Systemic arterial pressure, heart rate, and right ventricular systolic pressure (RVSP) were recorded and analyzed using a PowerLab 8/35 A/D converter data acquisition system (Chart, AD Instruments, Colorado Springs, CO, USA). After data acquisition, the pulmonary artery was removed, and the animals were euthanized.

### Echocardiography

The echocardiographic studies were performed using a murine-dedicated system (Vevo 2100^®^ equipment—Visualsonics^®^, Toronto, Canada) with a 40-mHz transducer, in 4 months HFD mice, as previously described (19, 20). Anesthesia was performed by placing the animal in a closed chamber ventilated with a titratable mixture of oxygen and isoflurane. The mouse was then placed on a 40°C-heated platform in the dorsal decubitus position, with continuous provision of isoflurane and shaved left hemithorax. Heart and respiratory rates were recorded continuously throughout the study. High-quality RV outflow pulsed-wave Doppler echocardiography was obtained from parasternal short-axis view 2D echocardiography. The following variables were measured: pulmonary acceleration time (PAT, measured from the beginning of RV outflow to the peak in ms) and RV ejection time (ET, the time from the onset to the end of RV outflow, in ms). The PAT/ET correlation ratio was calculated as described by Thibault et al. (21). Three cardiac cycles were analyzed and averaged for each measurement.

### RNA Extraction and Quantitative PCR

The messenger RNA (mRNA) level was determined in the isolated pulmonary artery. After total RNA purification and complementary DNA (cDNA) synthesis, quantitative PCR (qPCR) was performed as previously described (22). Specific primers were used for amplification of ATF6, Ire-1α, CHOP, PERK, PPAR-γ, and eIF2α, adiponectin genes; glyceraldehyde 3-phosphate dehydrogenase (GAPDH) and β-actin were used as endogenous control genes. The results were expressed as relative expression values compared to the respective CTL group.

### Statistical Analysis

All analyses were run in triplicate, and the results were expressed as mean ± standard deviation (SD); the number of animals used in each experiment are described in the figure legend. Differences between means were first analyzed by Student’s t-test or one-way ANOVA, followed by the *post-hoc* comparisons with Bonferroni test, where necessary (*p* < 0.05). The multiple comparisons test was used when appropriate. Statistical analysis was carried out with GraphPad Prism v.7.00 (GraphPad Software, San Diego, CA, USA).

## Results

### Characterization of Diet-Induced Insulin-Resistant Mice Model

**Figure 1:**
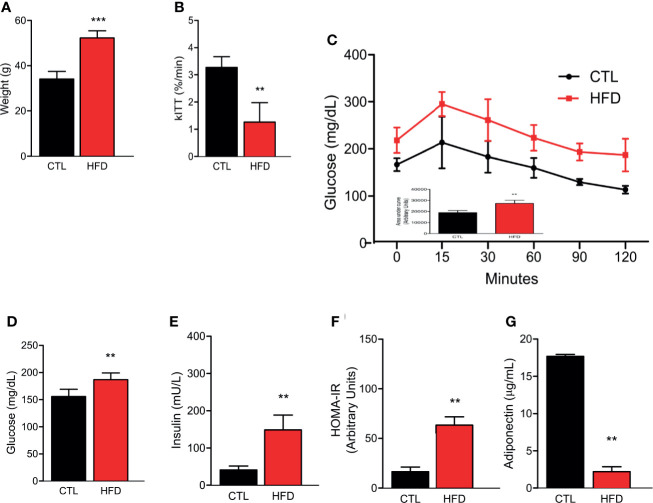
**(A)** Representative graphics of body weight in CTL mice (CTL, n = 8) and HFD mice fed (HFD n = 10). **(B)** kITT (CTL n = 5 and HFD n = 5). **(C)** Curve and AUC during the GTT (CTL n = 5 and HFD n = 7). **(D)** Fasting blood glucose (CTL n = 5 and HFD n = 5). **(E)** Fasting serum insulin (CTL n = 5 and HFD n = 5). **(F)** HOMA-IR calculated from the fasting concentrations of glucose (mg/dl) and insulin (mU/L) (CTL n = 5 and HFD n = 5). **(G)** Adiponectin levels (CTL n = 5 and HFD n = 9). Values represent mean ± SD. **p < 0.01 *vs.* CTL; ***p < 0.001 *vs.* CTL.

Previous data demonstrate that IR seems to increase the risk of PH (11, 23). Despite this, no study has been reported in the literature that shows the effect of a high-fat diet (HFD) and a well-described obese IR model, in the development of PH. In this regard, herein, we assessed the PH after 4 months of HFD. Before measuring PH, we first confirmed IR in our mice model. We observed that HFD group exhibited significant weight gain compared to animals fed on chow diet, i.e., the control group (CTL) (**Figure 1A**). The high-fat fed animals also presented superior insulin and glucose intolerance, as evidenced by lower constant for insulin tolerance test (kITT) and higher area under the curve (AUC) in the glucose tolerance test (GTT) when compared to their CTLs (**Figures 1B, C**). Additionally, high-fat feeding resulted in elevated fasting blood glucose and insulin levels, along with higher homeostasis model assessment insulin resistance (HOMA-IR) compared to lean animals (**Figures 1D–F**). Lastly, we measured the circulating levels of adiponectin, an adipokine that is secreted by adipose tissue and shows an inverse correlation to the amount of adipose tissue, and here, we show a decrease in adiponectin levels in HFD group when compared to CTL group (**Figure 1G**).

### High-Fat Diet for 4 Months Presents Slight Effects on PH

After confirming both obesity and IR status, the next step was to perform high-resolution transthoracic echocardiography and catheterization in CTL and mice with obesity. According to a validated study on echocardiographic measurements using catheterization (21), pulmonary acceleration time (PAT) values below 21 ms or PAT/ejection time (ET) ratio lower than 39% correlates with increased RVSP and, consequently, PH. Thus, the lower PAT/ET ratio, the higher the values of RVSP.

**Figure 2:**
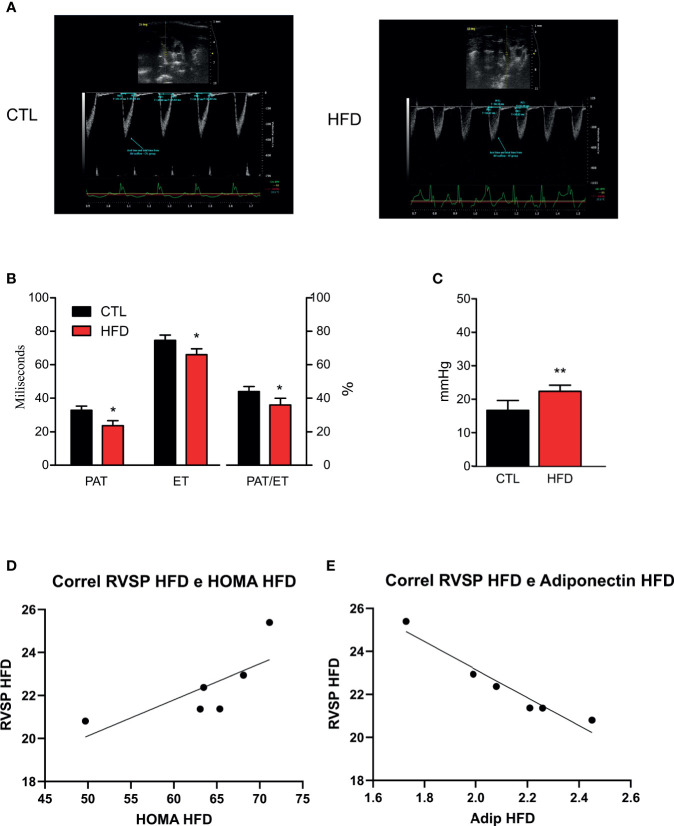
**(A)** Representative images and quantification graphics **(B)** of PAT, ET, and PAT/ET ratio obtained by RV outflow pulsed-wave Doppler echocardiography from CTL mice (CTL, n = 12) and HFD mice (HFD n = 15). **(C)** RVSP obtained by catheterization from CTL mice (n = 5) and HFD mice (n = 6). **(D)** Correlation of RVSP/HOMA-IR in HFD mice (n = 6). **(E)** Correlation of RVSP/adiponectin in HFD mice (n = 6). Values represent mean ± SD. *p < 0.05 *vs.* CTL, **p < 0.01 *vs.* CTL.

In the current study, we hypothesized that the measurements obtained by catheterization would be similar to those reported in models induced by monocrotaline with RVSP values >25 mmHg (24–26). Indeed, animals fed on an HFD displayed a more pronounced decrease in PAT than the ET, which in turn resulted in significant reduction in the PAT/ET ratio (**Figures 2A, B**). These data are directly related to the values of pulmonary arterial pressure obtained through catheterization. Regarding RVSP, we saw a significant increase in all HFD animals when compared to their respective CTL group (**Figure 2C**). Although not significant because of the small number of animals studied, there was a clear tendency to have a correlation between RVSP and HOMA-IR in HFD mice (r = 0.7434 and p = 0.0903) (**Figure 2D**). Furthermore, there was a significant correlation between RVSP and adiponectin (r = −0.676 and p = 0.0016) (**Figure 2E**).

### PH in HFD-Fed Mice Is Mediated by the Decrease in PPAR-γ Expression and Protein Levels in Pulmonary Artery

**Figure 3:**
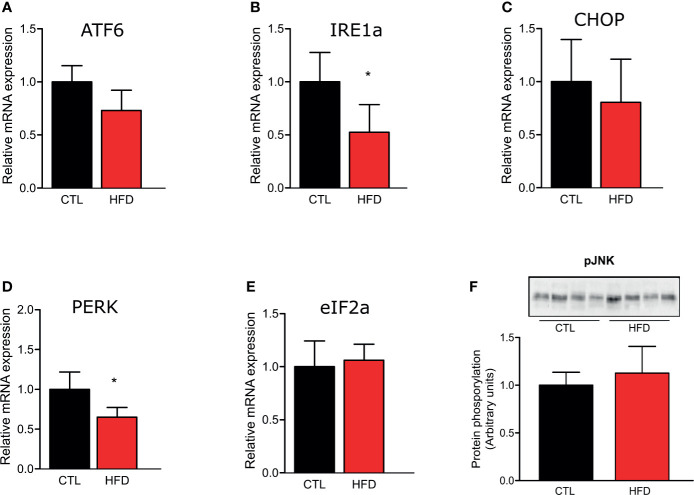
Representative graphics of ER stress markers relative gene expression, **(A)** ATF6, **(B)** IRE1, **(C)** CHOP, **(D)** PERK, and **(E)** eIF2α of the pulmonary artery from CTL mice (n = 5) and HFD mice (n = 5). **(F)** Protein amount of phosphorylated JNK protein of the pulmonary artery from CTL mice (n = 4) and HFD mice (n = 4). Values represent mean ± SD. *p < 0.05 *vs.* respective CTL.

To investigate if the molecular mechanism of PH could be associated to the deleterious effects of obesity, we evaluated the gene expression of ER stress markers ATF6, Ire1α, CHOP, PERK, and eIF2α in the pulmonary artery of CTL and HFD animals fed for 4 months (**Figures 3A–E**). We also observed JNK protein phosphorylation in CTL and HFD mice (**Figure 3F**). As shown in **Figure 3**, the results do not indicate a clear participation of ER stress in the PH observed in the mice with obesity.

**Figure 4:**
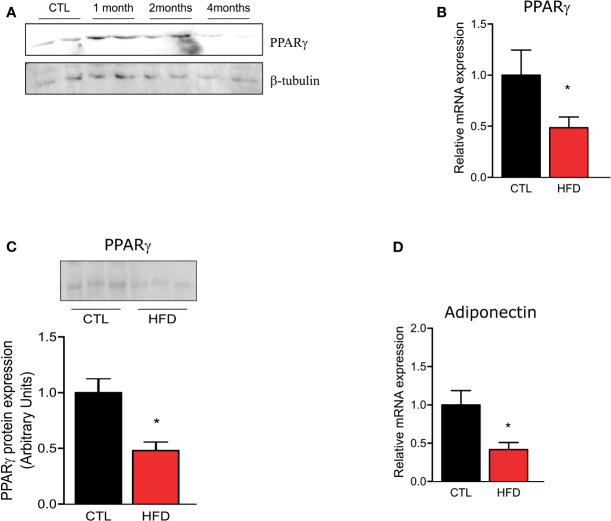
**(A)** Representative Western blots of PPARγ and β-tubulin from the pulmonary artery in CTL mice (n = 4) after 1, 2, or 4 months of HFD (n = 4). **(B)** Representative graphics of relative gene expression of PPAR-γ and of **(C)** PPAR-γ protein amount and **(D)** of relative gene expression of adiponectin from the pulmonary artery from CTL mice (n = 5) and HFD mice (n = 5). Values represent mean ± SD. *p < 0.05 *vs.* respective CTL.

Since PPAR-γ is reduced in lungs of patients who develop PH (12), we investigated if HFD fed mice presented a reduction in PPAR-γ gene and protein expression in the pulmonary artery. As evidenced in **Figure 4A**, PPAR-γ protein level shows an increase after 1 month of HFD returning to control levels at 2 months of HFD and decreasing at 4 months of HFD. Furthermore, at 4 months, PPAR-γ mRNA and protein levels are decreased in HFD mice (**Figures 4B, C**). Additionally, adiponectin mRNA, a marker of PPAR-γ activity, is also decreased in HFD mice when compared to CTLs (**Figure 4D**). In accordance with previous data (27), PPAR-γ protein levels were reduced in liver and muscle of HFD after 2 months of this diet but not in adipose tissue, and this reduction was maintained at 4 months of HFD (data not shown).

### PPAR-γ Agonist Treatment Improves PH in HFD Mice

**Figure 5:**
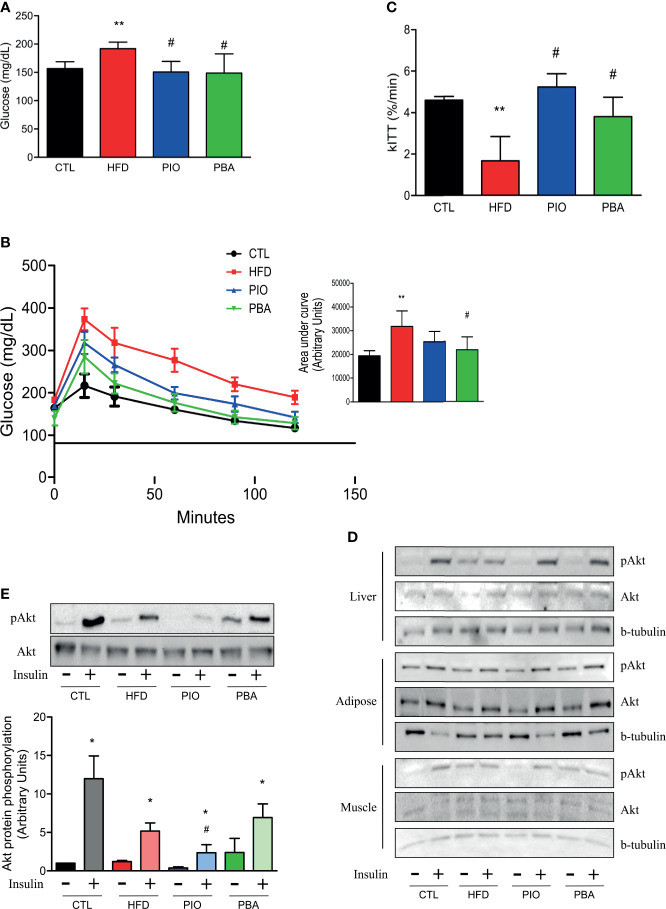
**(A)** Fasting blood glucose, **(B)** curve and AUC during the GTT, and **(C)** kITT from CTL mice (n = 5), HFD mice (n = 6), HFD mice treated with PIO (n = 5), and HFD mice treated with PBA (n = 5). **(D)** Western blot of phosphorylated Akt protein with or without insulin stimulation in liver, adipose tissue, and skeletal muscle from CTL mice (n = 4), HFD mice (n = 4), HFD mice treated with PIO (n = 4), and HFD mice treated with PBA (n = 4). **(E)** Western blot of phosphorylated Akt protein with or without insulin stimulation in the pulmonary artery from CTL mice (n = 4), HFD mice (n = 4), HFD mice treated with PIO (n = 4), and HFD mice treated with PBA (n = 4). Values represent mean ± SD. *p < 0.05 vs. same group without insulin, **p < 0.01 *vs.* CTL, ^#^p < 0.05 *vs.* HFD.

Since PPAR-γ and adiponectin mRNA levels are decreased in the pulmonary artery, and adiponectin circulating levels are also low in mice with obesity, we treated these mice with an agonist of PPAR-γ, PIO. PIO treatment was able to decrease HFD fasting blood glucose (**Figure 5A**) and also improve glucose and insulin tolerance, as evidenced by lower AUC of the GTT and higher kITT (**Figures 5B, C**). Interestingly, when we analyzed insulin signaling through Akt phosphorylation, we observed that PIO treatment was not able to reverse insulin signaling in the artery of mice with obesity (**Figures 5D, E**), even though it clearly improved whole-body insulin sensitivity.

**Figure 6:**
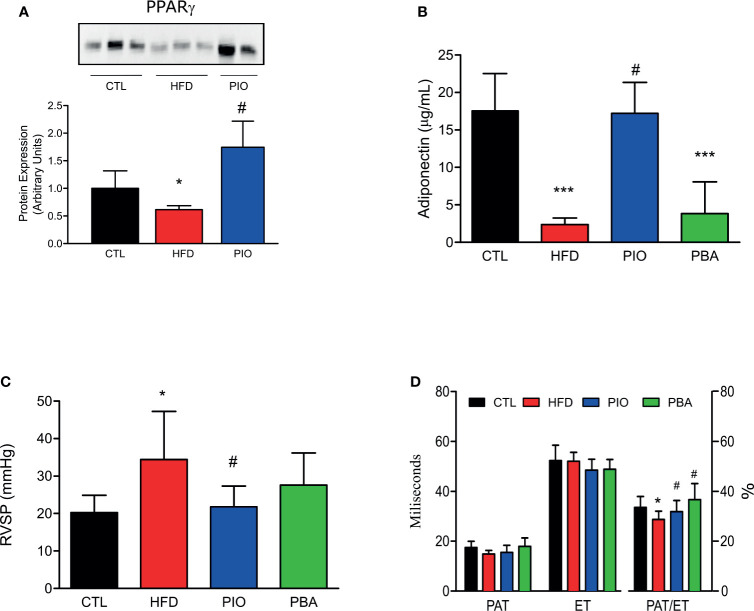
**(A)** Western blot of PPAR-γ in the pulmonary artery from CTL mice (n = 4), HFD mice (n = 4), HFD mice treated with PIO for 30 days (n = 4). **(B)** Circulating adiponectin levels from CTL mice (n = 4), HFD mice (n = 4), HFD mice treated with PIO for 30 days (n = 4), and HFD mice treated with PBA for 30 days (n = 4), **(C)** RVSP obtained by catheterization, and of PAT, ET, and PAT/ET ratio obtained by RV outflow pulsed-wave Doppler echocardiography from CTL mice (CTL, n = 6), HFD mice (n = 6), HFD mice treated with PIO for 30 days (n = 6), and HFD mice treated with PBA for 30 days (n = 5). **(D)** Quantification graphics of PAT, ET, and PAT/ET ratio obtained by RV outflow pulsed-wave Doppler echocardiography from CTL mice (n = 5) and HFD mice (HFD n = 5), HFD mice treated with PIO for 30 days (n = 5), and HFD mice treated with PBA for 30 days (n = 5). Values represent mean ± SD. *p < 0.001 *vs.* CTL, ***p < 0.001 *vs.* CTL and ^#^p < 0.05 *vs.* HFD.

Furthermore, PIO treatment was able to increase the PPAR-γ protein amount when compared to HFD (**Figure 6A**). Additionally, circulating adiponectin levels are also increased after PIO treatment (**Figure 6B**). We analyzed the measurements obtained by catheterization and observed that PIO treatment was able to decrease the RVSP to levels similar to CTL mice (**Figure 6C**); these results are also confirmed by the echocardiogram data (**Figure 6D**) that show an increase in the PAT/ET in PIO treated mice when compared to HFD.

To further verify the role of ER stress in our model, we also treated mice with an inhibitor of ER stress, PBA. Similar to PIO treatment, PBA was able to ameliorate some aspects of glucose and insulin intolerance, with decreased fasting blood glucose (**Figure 5A**), lower AUC in the GTT (**Figure 5B**), and increased kITT (**Figure 5C**). In addition, we also investigated the role of ER stress in insulin resistance in different tissues, through blockage of this phenomenon with PBA. The results showed that PBA improved insulin signaling in liver and adipose tissue but not in muscle (**Figure 5D**), and in pulmonary artery, this improvement is only mild (**Figure 5E**), pointing that ER has minimal or no role in insulin resistance in these last two tissues. However, PBA was not able to increase adiponectin levels (**Figure 6B**), and for this reason, no difference was observed in RVSP in PBA-treated mice when compared to HFD mice (**Figure 6C**), although echocardiogram results show an increase in PAT/ET (**Figure 6D**).

## Discussion

In the past 50 years, experimental and clinical studies have clearly shown a link between IR and cardiovascular disease. Although this link related to PH was previously suggested (28, 29), our report is the first experimental demonstration that HFD-induced IR, by itself, is accompanied by sustained PH established after 4 months of this diet. Previous data have shown that apoE-knockout mice on an HFD developed PH; clearly, these animals also developed an evident level of pulmonary atherosclerosis, which may be a bias in the correlation of IR with PH (12). Our model of obesity-induced IR showed an increase in pulmonary artery pressure, with an RVSP variation of 6–9 mmHg and also confirmed after catheterization, which was not based on left ventricular dysfunction and seems to be similar to other PH animal models induced by other methods (30, 31).

The molecular mechanisms of IR in liver, adipose tissue, and vessels in obesity can be unified through ER stress, which also accompanied different triggers of PH. More than 30 years ago, electron microscopy studies of PH tissues showed abnormal ER structure, suggesting that ER stress may have a pathophysiological role in this vascular alteration. More recently, the protein Nogo-B, a regulator of ER structure, was shown to be involved in PH through disruption of an ER-mitochondria unit and apoptosis suppression (13). However, in our model of obesity-induced PH, we did not find ER stress in the pulmonary artery, investigated through expression of PERK, IRE-1, and CHOP, suggesting that in this model of PH, ER stress may not be involved. Additionally, our data showed that ER stress has a tissue-specific regulation in insulin resistance, with an important role in liver and adipose tissue, but not in muscle, and only a mild or no role in pulmonary artery. Reinforcing these data, the treatment of mice with PBA, which attenuate systemic ER stress, mildly improved systemic IR and insulin signaling in the pulmonary artery but did not change PH. These data indicate that PH in obesity may be independent of ER stress and also of insulin signaling in the pulmonary artery.

Obesity and IR have complex interplay, and previous data have shown that in obesity, the IR may have a tissue-specific regulation and, in some situations, a pathway-specific defect (32–38), indicating that other pathways might be involved in the association between IR and PH. It is important to mention that systemic insulin resistance evaluated through HOMA showed a tendency of correlation with pulmonary artery hypertension in HFD mice. In this regard, we can suggest that the reduction in adiponectin levels in HFD mice may have an important role in this association. Reduced levels of adiponectin are uniformly observed in IR, and increased levels of this hormone reverse IR and reduce the risk of developing type 2 diabetes mellitus (39). Moreover, our data showed a clear inverse correlation between adiponectin levels and pulmonary hypertension. The reversal of IR in HFD mice treated with PIO, associated with an increase in circulating adiponectin and partial improvement in PH, suggests that this hormone may directly contribute to the reduction in pulmonary artery pressure. In addition to circulating levels, the reduced expression of adiponectin in perivascular adipose tissue of the pulmonary artery was also reversed by PIO, suggesting a possible paracrine effect of this adipocytokine. Adiponectin is able to reduce the mitogenic function of vascular smooth cells, possibly through reduction in PDGF-BB and also to suppress intimal thickening.

Moreover, one of the most important findings of our data is the reduction in PPAR-γ RNA and protein expression in the pulmonary artery in obesity-induced PH, which are both reversed by PIO. Right ventricular heart failure is the convergent cause of death in most patients with PH. Previous data showed that PPAR-γ has a vasoprotective role in smooth muscle cells and also endothelial cells, probably through a metabolic regulation (40) and that deletion of PPAR-γ in cardiomyocytes induces intramyocellular lipid accumulation and systolic dysfunction in both ventricles. In another PH model, the SU5416/hypoxia (SuHx) rat model, there is lipotoxicity in the heart associated with epigenetic, transcriptional, and metabolic alterations (16). In this model, it was demonstrated that PIO can normalize lipid metabolism and mitochondrial dysfunction, reversing the epigenetic and transcriptional alterations and improving right ventricular (RV) failure and PH.

Previous data showed that loss-of-function mutations in the BMP-RII gene frequently occur in cases of familial and idiopathic PH and that this alteration would decrease endogenous PPAR-γ activity (6, 41). It is important to mention that BMP-2 may activate PPAR-γ. In this regard, the activation of PPAR-γ by PIO may at least partially reverse PH in these patients. More importantly, BMPR-II is a dominant gene with low penetrance, resulting in only 20% of affected family members developing the disease, reinforcing the important role of acquired factors such as obesity and IR to potentiate BMPR-II mutations.

## Conclusions

In summary, the results of the present study demonstrated that a HFD for at least 4 months is able to increase pulmonary artery pressure, and this animal model can be used to investigate the link between IR and PH. In this model, there was a reduction in circulating adiponectin and in perivascular adiponectin expression in the pulmonary artery, associated with a reduction in PPAR-γ expression in this artery. Treatment with PIO improved IR and PH and reversed the lower expression of adiponectin in perivascular and PPAR-γ in the pulmonary artery, highlighting this drug as potential benefit for this poorly recognized complication of obesity.

## Data Availability Statement

The raw data supporting the conclusions of this article will be made available by the authors, without undue reservation.

## Ethics Statement

The animal study was reviewed and approved by Comissão de Ética no Uso de Animais/Instituto de Biologia/Universidade Estadual de Campinas.

## Author Contributions

AG, AO, and GR: researched data, contributed to the discussion, and wrote/reviewed/edited manuscript. RM, RC, AS, HC, DG, and ZM: researched data and reviewed/edited manuscript. OP, VS, and MS contributed to the discussion and wrote/reviewed/edited manuscript. All authors contributed to the article and approved the submitted version.

## Funding

This research was supported by INCT de Obesidade e Diabetes 465693/2014-8 (FAPESP/CNPq) and postdoctoral fellowship 2012/02669-2 (FAPESP) ad CAPES.

## Conflict of Interest

The authors declare that the research was conducted in the absence of any commercial or financial relationships that could be construed as a potential conflict of interest.

## Publisher’s Note

All claims expressed in this article are solely those of the authors and do not necessarily represent those of their affiliated organizations, or those of the publisher, the editors and the reviewers. Any product that may be evaluated in this article, or claim that may be made by its manufacturer, is not guaranteed or endorsed by the publisher.

## Supplementary Material

The Supplementary Material for this article can be found onlineat: https://www.frontiersin.org/articles/10.3389/fendo.2021.701994/full#supplementary-material

Supplementary Figure 1 Cartoon representing experimental design for both set of experiments. **(A)** Four months of standard rodent chow (CTL) versus high-fat diet (HFD) treatment. **(B)** Four months of standard rodent chow (CTL), high-fat diet (HFD), high-fat diet with pioglitazone during the last month (PIO) and high fat diet with 4-phenylbutyric acid during the last month (PBA).
