# Supplementary figures and images for: Pulmonary Hypertension in Obese Mice Is Accompanied by a Reduction in PPAR-γ Expression in Pulmonary Artery
Source: Front Endocrinol (Lausanne). 2021 Sep 6;12:701994. doi: 10.3389/fendo.2021.701994 (PMC8450870; doi:10.3389/fendo.2021.701994)

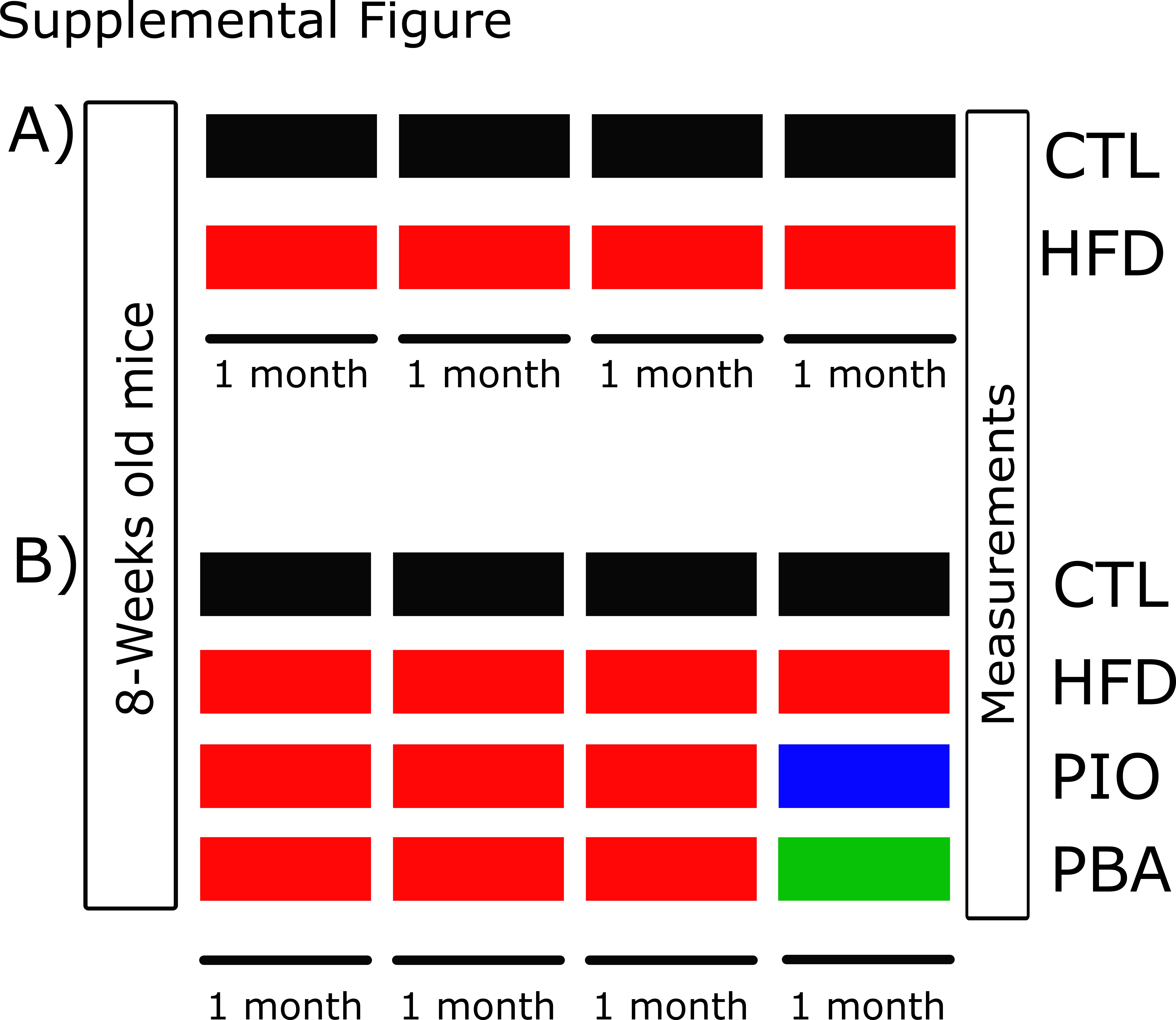

Supplement: Supplementary Figure 1 — Cartoon representing experimental design for both set of experiments. (A) Four months of standard rodent chow (CTL) versus high-fat diet (HFD) treatment. (B) Four months of standard rodent chow (CTL), high-fat diet (HFD), high-fat diet with pioglitazone during the last month (PIO) and high fat diet with 4-phenylbutyric acid during the last month (PBA). [file Image_1.jpeg]
